# Increasing anti-S antibody testing: a quality improvement initiative with evolving COVID-19 guidelines

**DOI:** 10.1136/bmjoq-2022-001886

**Published:** 2022-09-13

**Authors:** Ali M Alam, Rebecca Lester, Marie-Claire Hoyle, Tom E Fletcher, Paul Hine

**Affiliations:** 1 Institute of Infection, Veterinary and Ecological Sciences, University of Liverpool, Liverpool, UK; 2 Tropical and Infectious Diseases Unit, Royal Liverpool University Hospital, Liverpool, UK; 3 Liverpool School of Tropical Medicine, Liverpool, UK

**Keywords:** COVID-19, Quality improvement, Quality measurement

## Abstract

**Background:**

COVID-19 management guidelines are constantly evolving, making them difficult to implement practically. *Ronapreve* was a neutralising monoclonal antibody introduced into UK COVID-19 guidelines in 2021. It reduces mortality in seronegative patients infected with non-omicron variants. Antibody testing on admission is therefore vital in ensuring patients could be considered for *Ronapreve* as inpatients.

**Local problem:**

We found that on our COVID-19 ward, 31.4% of patients were not having anti-S tests despite fulfilling the other criteria to be eligible for *Ronapreve*. This was identified as an important target to improve; by not requesting anti-S tests, we were forgoing the opportunity to use an intervention that could improve outcomes.

**Methods:**

We analysed patient records for patients with COVID-19 admitted to our ward over 4 months to observe if awareness of the need to request anti-S increased through conducting plan–do–study–act (PDSA) cycles.

**Interventions:**

Our first intervention was an multidisciplinary team (MDT) discussion at our departmental audit meeting highlighting our baseline findings and the importance of anti-S requesting. Our second intervention was to hang printed posters in both the doctors’ room and the ward as a visual reminder to staff. Our final intervention was trust-wide communications of updated local COVID-19 guidance that included instructions for anti-S requesting on admission.

**Results:**

Our baseline data showed that only 68.6% of patients with symptomatic COVID-19 were having anti-S antibody tests requested. This increased to 95.0% following our three interventions. There was also a reduction in the amount of anti-S requests being ‘added on’, from 57.1% to 15.8%.

**Conclusions:**

COVID-19 guidelines are constantly evolving and require interventions that can be quickly and easily implemented to improve adherence. Sustained reminders through different approaches allowed a continued increase in requesting. This agrees with research that suggests a mixture of educational sessions and visual reminders of guidelines increase their application in clinical practice.

## Introduction

COVID-19 is a major healthcare issue in the UK with over 720 000 patients admitted to hospital since the start of the pandemic.^1^ Large-scale trials have led to the identification of novel drugs that can reduce the severity of COVID-19.^2^

In August 2021, casirivimab/imdevimab (*Ronapreve*) received authorisation for use in the treatment of COVID-19 in the UK.^3^ This medication has been shown to reduce the mortality of COVID-19 by 20%.^4 5^ Importantly, the trial data support its use in seronegative patients infected with non-omicron variants, making antibody testing paramount for optimising its allocation.^6^ Antibody testing is conducted through requesting a serum COVID-19 anti-S antibody (anti-S) test.

As a novel disease, COVID-19 guidelines are constantly changing, resulting in poor practical implementation.^7^ In September 2021, UK COVID-19 management guidelines stated that all patients hospitalised for acute COVID-19 illness should be considered for *Ronapreve,* and therefore should receive an anti-S test.^8^ Ideally, testing should be done on admission to allow earlier administration of *Ronapreve* (if indicated).

Despite these national guidelines, more than 30% of the patients with COVID-19 on our ward were not having anti-S tests. This was identified as an important target to improve; by not requesting anti-S tests, we were forgoing the opportunity to use an intervention that improves outcome in a subset of patients.

The ward we studied is a 13-bed isolation unit in a city centre hospital that is staffed by a consultant and two junior clinicians. Both the clinicians and the nurses on the ward request serum tests and the project team identified both teams as targets of our interventions.

Our aim was to ensure all patients on our ward received an anti-S antibody test after admission for symptomatic COVID-19 within a 4-month intervention period.

## Methods

We analysed patient records for all COVID-19 patients admitted to our ward over a 4-month period. Data were collected at weekly intervals, and patients were subsequently grouped into the month of their admission.

We chose to study the binary outcome of whether anti-S tests were requested for patients on the ward (‘Anti-S test requested’). We used this as a measure of awareness of the *Ronapreve* guidelines. We also gathered information whether these anti-S requests had been placed through ‘adding on’ the test; this occurs when the lab is phoned and requested to run the test on an old serum sample. We expected that as awareness of guidelines increased, more anti-S tests would be taken in blood samples on admission to the ward and so the need to add-on the test would decrease. Both these outcomes were measured by retrospectively reviewing ICE (CliniSys Group, UK) request data for our patients.

We illustrated the performance of anti-S requesting through a line chart. The descriptive variables we collected included age and sex.

## Plan–do–study–act (PDSA) cycles

We conducted three PDSA cycles within our study period (online supplemental file 1). Our objective was that by February 2022, every eligible patient had an anti-S test requested on admission through implementing fast and feasible interventions.

10.1136/bmjoq-2022-001886.supp1 Supplementary data

Before we began planning our interventions, discussion with our ward staff illustrated that there was poor understanding of *Ronapreve* guidelines and why anti-S requesting is of importance. Though consultants had received emails regarding the updated COVID-19 guidelines, this had not been disseminated to junior staff. We therefore identified awareness of guidelines as a root cause of why patients were not receiving anti-S requests.

Our first intervention was an educational session at our departmental audit meeting highlighting our baseline findings and the importance of anti-S requesting. We believed this would increase clinicians’ knowledge about the guidelines at the time and highlight a gap in our treatment provision. We expected an increase in requesting following this meeting and collected data a month after our first intervention.

Though we saw an increase in requesting following the first intervention, we believe the intervention failed to educate two vital groups: nurses and clinicians who did not attend our audit meeting. Our second intervention was therefore to hang printed posters with instructions to order anti-S tests in both the doctors’ room and the ward as a visual reminder to all staff on the ward. We hypothesised that this would lead to a substantial increase in anti-S requesting.

Healthcare workers based on COVID-19 wards have higher levels of stress, anxiety and burnout compared with those working in other wards,^9^ and to help alleviate this, clinicians in our hospitals rotate between COVID-19 wards and other wards. After collecting data following our second intervention, we realised that rotational staff may not be educated in the *Ronapreve* guidelines. Therefore, we planned our final intervention to include a trust-wide email update of its COVID-19 guidance with instructions for anti-S requesting on admission.

## Results

The total number of patients studied in our cohort was 140. The mean age was 60.6 years (SD±19.3), and 67 (47.9%) patients were female (online supplemental file 2).

**Figure 1:**
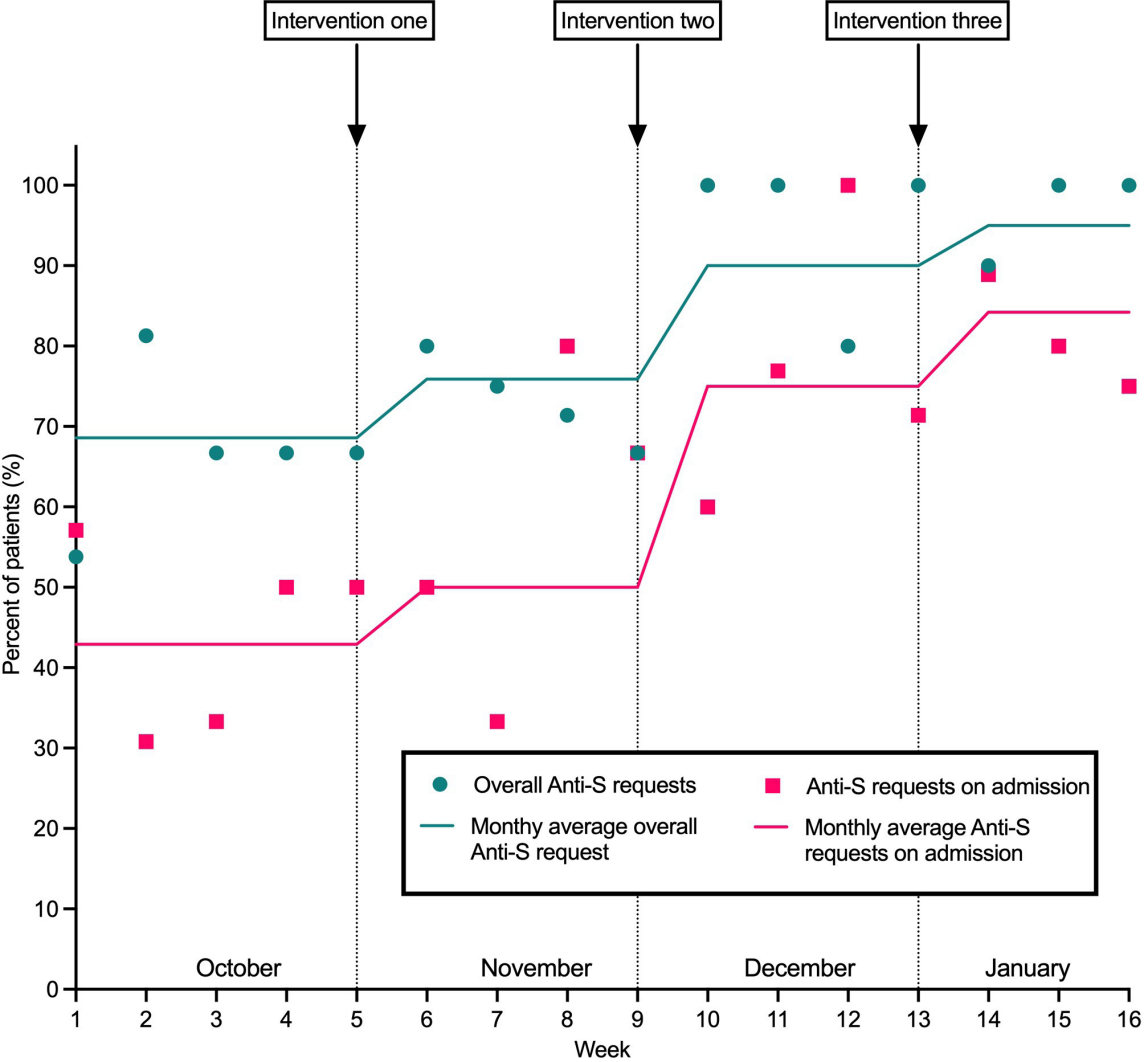
Line graph showing change in total anti-S requesting and on admission anti-S requesting throughout our study period.

Our baseline data showed that only 68.6% of patients with symptomatic COVID-19 were having anti-S antibody tests requested. This increased to 95.0% by January following the three interventions (figure 1). A month after the first intervention, requesting increased by 7.9%. Our second intervention showed the greatest monthly increase in requesting at 14.1%. The final intervention led to an increase of 5.0%. There was also a reduction in the amount of anti-S requests being added on, from 57.1% to 15.8% (online supplemental file 3).

## Discussion

Guidelines are ubiquitous in healthcare and adherence to these can improve patient outcomes.^10^ Multiple factors can influence guideline compliance, and studies on improving adherence may not be generalisable as barriers in one setting may not be present in another.^11^ We identified clinician lack of awareness as a substantial barrier with the *Ronapreve* guidelines. This issue is pertinent in the case of novel conditions like COVID-19, where best practice changes frequently as more management options are discovered.^7^

Overall, our results suggest there was greater understanding about the importance of anti-S antibody requesting over the 4-month intervention period. Our findings agree with research that has suggested a mixture of educational sessions and visual reminders increase application of guidelines in clinical practice.^12 13^ Though all our interventions increased requesting of anti-S tests, we found that the greatest increase occurred after placing posters in the ward. We feel this worked best as it acts as a simple and constant visual reminder to all staff on the ward. In comparison, our first MDT discussion led to a sharp weekly increase that then decreased, owing to the short-term effects of educational sessions. A weakness in this intervention was that we only presented to the clinical team at the departmental audit meeting. This intervention did not reach the nursing staff, which is vital as they often request admission bloods prior to clerking. This formed the basis of our second intervention involving posters. We hoped the final intervention of a trust-wide email would reach any staff members who had not been present for our first intervention and may have not seen the posters in the second intervention. This intervention led to only a small increase in requesting, likely due to our baseline requesting being at a high level and reasons such as staff not checking emails.

Limitations in our work include the fact that other interventions may have taken place during our study period. Second, there were cases with valid reasons for not requesting anti-S tests; for example, some patients refused as they would not like to be considered for monoclonal treatment, while some, though symptomatic, were well enough to go home on the day of admission and therefore did not have anti-S tests done. These cases, however, were not very prevalent on our ward.

We were not able to achieve our main aim to ensure that every symptomatic patient had anti-S requests. Our short intervention period may have been a factor in this, and our target may have been met if further rounds of data collection was possible. However, we could not collect further data to report the sustainability of our interventions as guidelines changed at the end of our quality improvement initiative. *Ronapreve* is ineffective against the omicron variant, and by the end of January, omicron was the dominant variant in the UK. Our initiative illustrates how fast and feasible interventions can be implemented to increase awareness of changing guidelines. We felt that if another novel treatment option was developed, we could use our findings as the basis of planned actions to increase awareness, and indeed, a similar approach was taken to improve awareness of *nirmatrelvir/ritonavir* and *sotrovimab,* which were both introduced to COVID-19 guidelines at the end of 2021.

## Conclusion

Our three PDSA cycles led to a material increase in anti-S antibody requesting. Our work suggests that a mixture of education and visual posters can act as simple and effective interventions to improve uptake and awareness of evolving guidelines.

## References

[1] United Kingdom Government. Patients admitted to hospital. Available: https://coronavirus.data.gov.uk/details/healthcare?areaType=overview&areaName=United%20Kingdom#card-patients_admitted_to_hospital [Accessed 21 Feb 2022].

[2] Randomised evaluation of COVID-19 therapy. results. Available: https://www.recoverytrial.net/results [Accessed 21 Feb 2022].

[3] Medicines & Healthcare products Regulatory Agency. Summary of product characteristics for Ronapreve. Available: https://www.gov.uk/government/publications/regulatory-approval-of-ronapreve/summary-of-product-characteristics-for-ronapreve [Accessed 21 Feb 2022].

[4] Department of Health & Social Care. Antibody testing for SARS-CoV-2: key information. Available: https://www.gov.uk/government/publications/antibody-testing-for-sars-cov-2-key-information/antibody-testing-for-sars-cov-2-information-for-general-practitioners [Accessed 21 Feb 2022].

[5] Randomised Evaluation of COVID-19 Therapy (RECOVERY). RECOVERY trial finds Regeneron’s monoclonal antibody combination reduces deaths for hospitalised COVID-19 patients who have not mounted their own immune response. Available: https://www.recoverytrial.net/news/recovery-trial-finds-regeneron2019s-monoclonal-antibody-combination-reduces-deaths-for-hospitalised-covid-19-patients-who-have-not-mounted-their-own-immune-response-1 [Accessed 21 Feb 2022].

[6] Sidebottom DB, Gill D. Ronapreve for prophylaxis and treatment of covid-19. BMJ 2021;374:n2136. 10.1136/bmj.n2136 34475117

[7] Saperstein Y, Ong SY, Al-Bermani T, et al. COVID-19 guidelines changing faster than the virus: implications of a clinical decision support APP. Int J Clin Res Trials 2020;5:148. 10.15344/2456-8007/2020/14832832740

[8] Department of Health & Social Care. Interim clinical commissioning policy: antivirals or neutralising monoclonal antibodies in the treatment of COVID-19 in hospitalised patients (version 5). Available: https://www.england.nhs.uk/coronavirus/wpcontent/uploads/sites/52/2021/09/C1560-ii-interim-ccp-antivirals-neutralising-monoclonal-antibodies-treatment-of-covid-19-in-hospitalised-patie.pdf [Accessed 21 Feb 2021].

[9] Tiete J, Guatteri M, Lachaux A, et al. Mental health outcomes in healthcare workers in COVID-19 and non-COVID-19 care units: a cross-sectional survey in Belgium. Front Psychol 2020;11:612241. 10.3389/fpsyg.2020.612241 33469439 PMC7813991

[10] Brennan C, Greenhalgh J, Pawson R. Guidance on guidelines: understanding the evidence on the uptake of health care guidelines. J Eval Clin Pract 2018;24:105–16. 10.1111/jep.12734 28370699

[11] Cabana MD, Rand CS, Powe NR, et al. Why don't physicians follow clinical practice guidelines? A framework for improvement. JAMA 1999;282:1458–65. 10.1001/jama.282.15.1458 10535437

[12] Fischer F, Lange K, Klose K. Barriers and strategies in guideline Implementation—A scoping review. Health Care 2016;4:36. 10.3390/healthcare4030036 27417624 PMC5041037

[13] Grol R, Grimshaw J. From best evidence to best practice: effective implementation of change in patients’ care. The Lancet 2003;362:1225–30. 10.1016/S0140-6736(03)14546-1 14568747

