# Supplementary material for: Increasing anti-S antibody testing: a quality improvement initiative with evolving COVID-19 guidelines
Source: BMJ Open Qual. 2022 Sep 13;11(3):e001886. doi: 10.1136/bmjoq-2022-001886 (PMC9471206; doi:10.1136/bmjoq-2022-001886)

Supplement 1 – Diagram illustrating our Plan-Do-Study-Act cycles

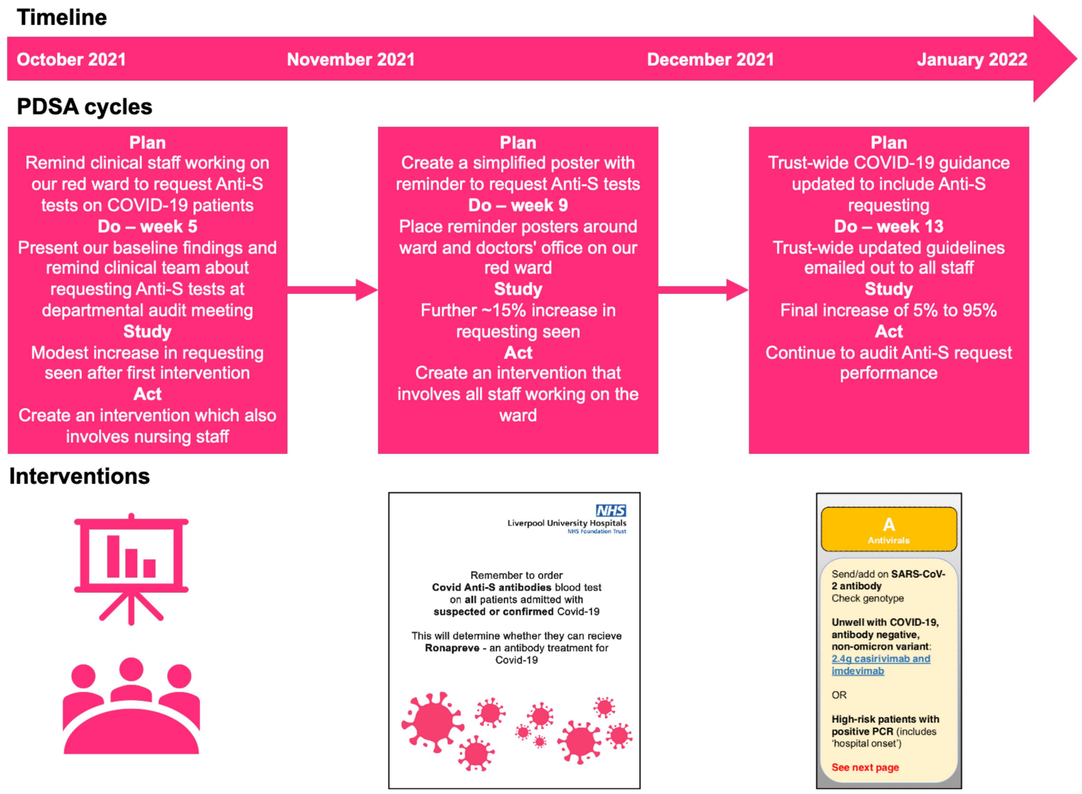

Supplement 2 – Characteristics and number of patients receiving Anti-S requests in our 4-month intervention period.

	Total	October	November	December	January
Admissions	140	51	29	40	20
Mean age (±SD)	60.6 (±19.3)	62.5 (±18.0)	56.8 (±21.6)	61.4 (±16.9)	59.9 (±23.7)
Female (%)	67 (47.9%)	18 (35.3%)	16 (55.1%)	20 (50.0%)	13 (65.0%)
Male (%)	73 (51.8%)	33 (64.7%)	13 (44.9%)	20 (50.0%)	7 (35.0%)
Anti-S test request (%)	112 (80.0%)	35 (68.6%)	22 (75.9%)	36 (90.0%)	19 (95.0%)

Supplement 3 – Graph illustrating the breakdown of Anti-S requests by month

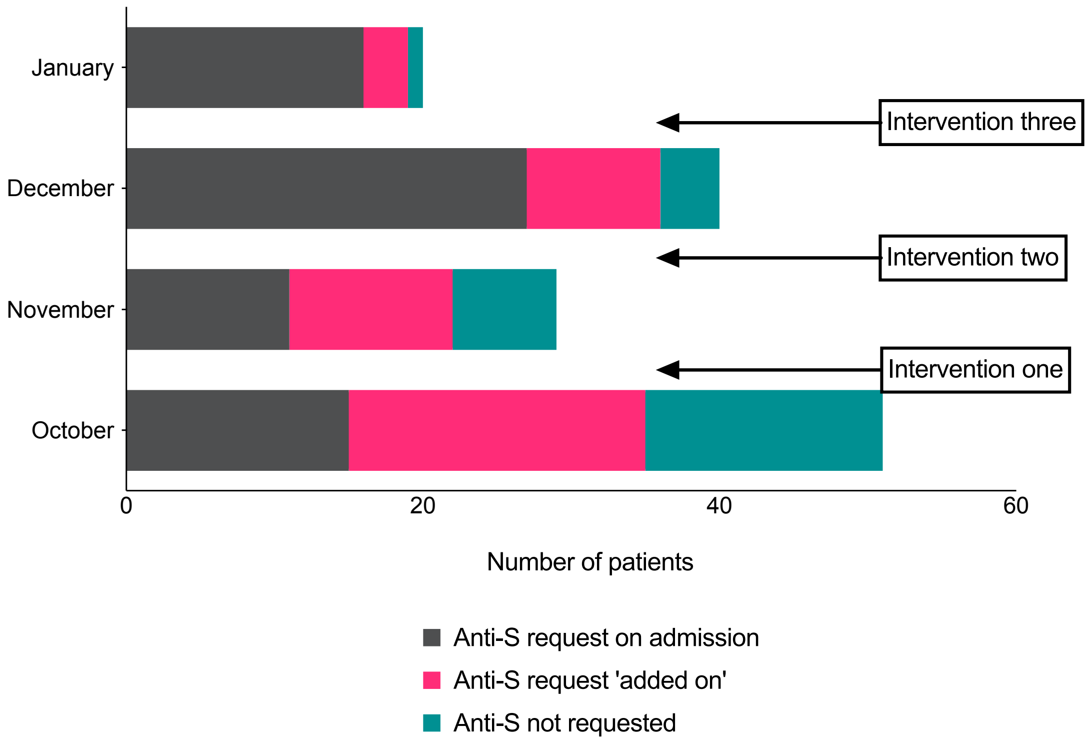

Supplement: Supplementary data [file bmjoq-2022-001886supp001.pdf]
